# LRP-1 and LRP-2 receptors function in the membrane neuron. Trafficking mechanisms and proteolytic processing in Alzheimer's disease

**DOI:** 10.3389/fphys.2012.00269

**Published:** 2012-07-16

**Authors:** Carlos Spuch, Saida Ortolano, Carmen Navarro

**Affiliations:** Department of Pathology and Neuropathology, University Hospital of VigoVigo, Spain

**Keywords:** Alzheimer's disease, astrocytes, amyloid-beta, intracellular domain, LRP-1, LRP-2, megalin, central nervous system, brain, neurodegenerative diseases, neuron

## Abstract

Low density lipoprotein receptor-related protein (LRP) belongs to the low-density lipoprotein receptor family, generally recognized as cell surface endocytic receptors, which bind and internalize extracellular ligands for degradation in lysosomes. Neurons require cholesterol to function and keep the membrane rafts stable. Cholesterol uptake into the neuron is carried out by ApoE via LRPs receptors on the cell surface. In neurons the most important are LRP-1 and LRP-2, even it is thought that a causal factor in Alzheimer's disease (AD) is the malfunction of this process which cause impairment intracellular signaling as well as storage and/or release of nutrients and toxic compounds. Both receptors are multifunctional cell surface receptors that are widely expressed in several tissues including neurons and astrocytes. LRPs are constituted by an intracellular (ICD) and extracellular domain (ECD). Through its ECD, LRPs bind at least 40 different ligands ranging from lipoprotein and protease inhibitor complex to growth factors and extracellular matrix proteins. These receptors has also been shown to interact with scaffolding and signaling proteins via its ICD in a phosphorylation-dependent manner and to function as a co-receptor partnering with other cell surface or integral membrane proteins. Thus, LRPs are implicated in two major physiological processes: endocytosis and regulation of signaling pathways, which are both involved in diverse biological roles including lipid metabolism, cell growth processes, degradation of proteases, and tissue invasion. Interestingly, LRPs were also localized in neurons in different stages, suggesting that both receptors could be implicated in signal transduction during embryonic development, neuronal outgrowth or in the pathogenesis of AD.

## The low-density lipoprotein receptor (LDLR) family

**Figure 1:**
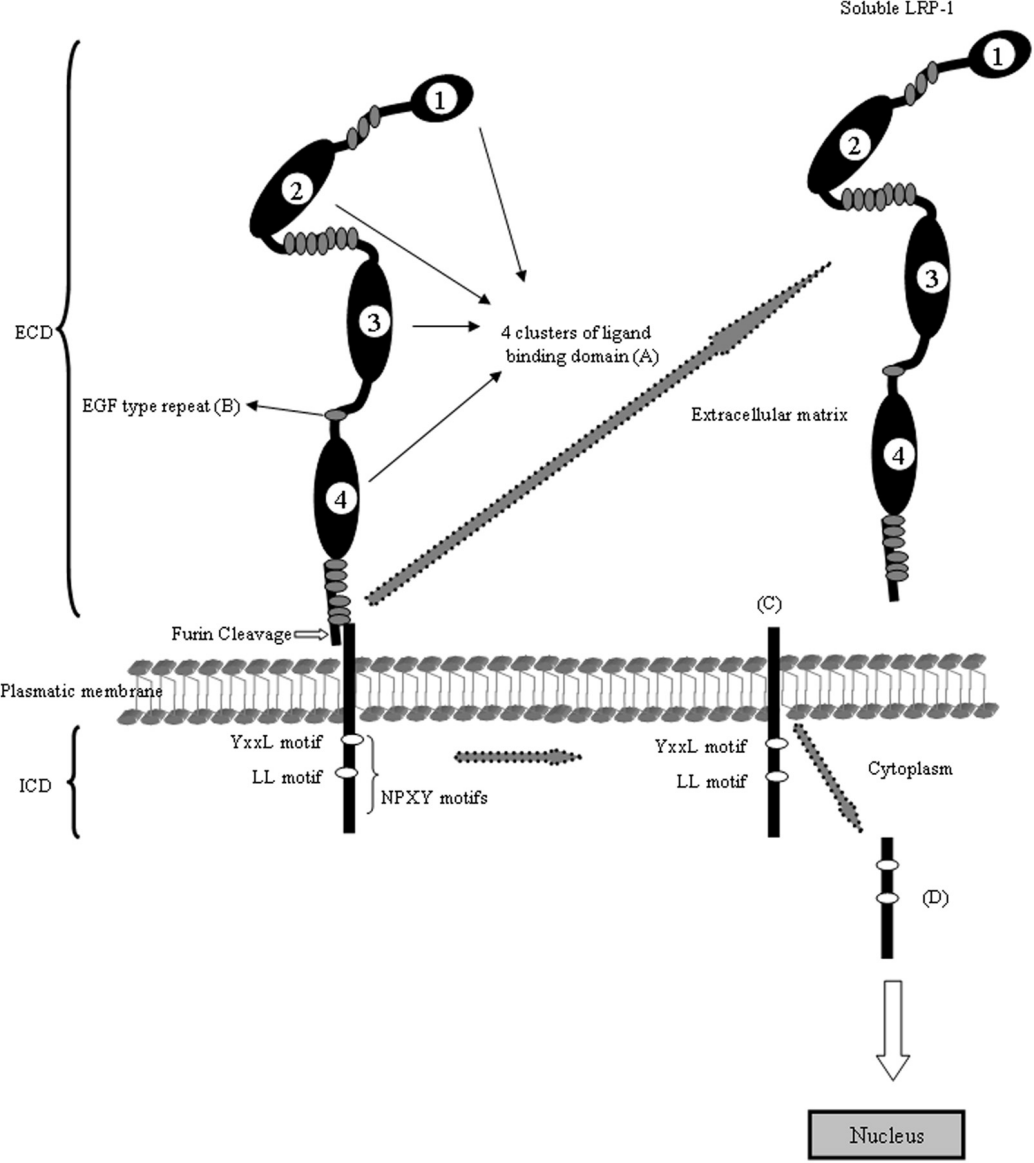
**Schematic domain organization of LRP1.** The LRP1 contains five different domains: **(A)** the ligand-binding domain, **(B)** the EGF-precursor homology domain, **(C)** the O-linked sugar domain, **(D)** the transmembrane domain and **(E)** the intracellular domain. The ligand-binding domain consists of four clusters and mediates the binding to ligands (see Table 1). The hydrophobic transmembrane domain ensures the anchoring the LRP1 in the plasma membrane. The cytoplasmic tail of the LRP1 containing the characteristic NPXY sequence interact with phospho-tyrosine binding domains of cellular adaptors proteins which are important for endocytosis and subsequent intracellular transport. The LRP1 is proteolytically cleaved within the Golgi complex to generate two subunits: **(A)** the N-terminal 515-kDa α-subunit containing the ligand-binding domains and **(B)** the C-terminal 85-kDa β-subunit containing an extracellular part, the transmembrane spanning domain and the cytoplasmic intracellular domain. The cytoplasmatic LRP1 α-subunit contains diverse potential endocytosis and signaling motifs: two NPXY motifs whereas the distal NPXY sequence overlaps with the endocytosis signal XYYL and two dileucine motifs. With arrows is indicating the cleavage events in the molecule and the resulting fragments.

The LDLR family consists of more than 11 receptors that function in receptor-mediated endocytosis and cellular signaling (Herz and Bock, 2002). In addition to the LDLR itself, the family includes LRP1 (Herz et al., 1988), LRP-2, also called megalin, (Spuch and Navarro, 2010a,b), VLDLR (Takakashi et al., 1992), LRP5 (Hey et al., 1998; Kim et al., 1998), LRP6 (Brown et al., 1998), ApoE receptor 2 (ApoER2), also called LRP8 (Kim et al., 1996; Novak et al., 1996; Brandes et al., 1997), sorLA-1, also called LR11, (Jakobsen et al., 1996; Yamazaki et al., 1996), LRP1B (Liu et al., 2000) and the most recently identified the LRAD3 (Ranganathan et al., 2011). A model depicting the major structural components of the representative receptors is shown in Figure 1.

### Low density lipoprotein receptor-related protein-1 (LRP1)

The LRP1, also known as CD91 or α2macroglobulin receptor, is a multifunctional scavenger and signaling receptor that belongs to the LRP family (Bruno et al., 2010; Boucher and Herz, 2011). LRP1 is a massive protein (600 kDa) that is proteolytically nicked during biosynthesis to give two stably associated polypeptides: an 85-kDa membrane-spanning C-terminal fragment and a 515-kDa extracellular N-terminal chain. The extracellular heavy α-chain of LRP1 is non-covalently coupled to the transmembrane and cytoplasmic light β-chain domain. The α-chain contains four ligand-binding domains (clusters 1–4), consisting of 2, 8, 10, and 11 cysteine-rich complement-type repeats, respectively (Obermoeller-McCormick et al., 2001) (Figure 1). The LRP1 ligand-binding domains 2 and 4 are the major LRP1 binding regions interacting with a diverse array of approximately forty structurally diverse ligands. LRP1 is expressed abundantly on neurons (Moestrup and Verroust, 2001; Kanekiyo et al., 2011), where its fundamental role is the uptake of cholesterol and fatty acids required for synapse formation (Mauch et al., 2001; Fester et al., 2009). In addition, LRP1 binds more than 30 ligands extracellularly, including ApoE, α2-macroglobulin, tissue plasminogen activator (tPA), proteinase-inhibitors, blood coagulation factors, receptor-associated protein (RAP), Aβ, prion protein and aprotinin (Hussain et al., 1999; Neels et al., 1999; Herz, 2001; Herz and Strickland, 2001; Croy et al., 2003; Deane et al., 2004a,b; Meijer et al., 2007; Demeule et al., 2008; Lillis et al., 2008; Parkyn et al., 2008) (Table 1). Interestingly, its cytoplasmic domain binds to endocytic and scaffold adaptors that link the receptor to other membrane proteins, including Amyloid Precursor Protein (APP) (Herz and Chen, 2006; Waldron et al., 2008). The cytoplasmic tail of LRP1 contains two NPXY motifs, one YXXL motif and two di-leucine motifs (Li et al., 2001). It has been suggested that these motifs may be associated with the rapid endocytotic rate of LRP1 (Deane et al., 2008). The cytoplasmic tail is phosphorylated on serine and/or tyrosine residues (van der Geer, 2002) and can interact with different adaptor proteins associated with cell signaling, such as disabled-1 (Dab1), FE65 (Klug et al., 2011) and postsynaptic density protein 95 (PSD95) (Trommsdorff et al., 1998; Gotthardt et al., 2000; Herz et al., 2009). Thus, LRP1 has a dual role as a receptor which internalizes its ligands acting like a rapid cargo endocytotic cellular transporter and also as transmembrane cell signaling receptor (Pflanzner et al., 2011).

**Table 1:** **Ligands of LRP1 and LRP2**.

**Receptor**	**Ligands**	**References**
LRP1 and LRP2	Albumin	Cui et al., 1996
LRP2	Aminoglycosides	Moestrup and Verroust, 2001
LRP2	α-Amilase	Birn et al., 2000
LRP1 and LRP2	Angiotensin II	Gonzalez-Villalobos et al., 2005
LRP1 and LRP2	Angiotensin 1–7	Gonzalez-Villalobos et al., 2006
LRP1 and LRP2	ApoB	Stefansson et al., 1995
LRP1 and LRP2	ApoE	Willnow, 1999
LRP1 and LRP2	ApoH	Moestrup and Verroust, 2001
LRP1 and LRP2	Apoj (Clusterin)	Kounnas et al., 1995; Hammad et al., 1997
LRP1 and LRP2	ApoM	Faber et al., 2006
LRP1 and LRP2	Aprotinin	Moestrup and Verroust, 2001
LRP1 and LRP2	Bone morphogenetic protein 4	Spoelgen et al., 2005
LRP1 and LRP2	Ca^2+^	Christensen and Nielsen, 2007
LRP1 and LRP2	Cathepsin b	Nielsen et al., 2007
LRP1 and LRP2	Coagulation Factor VIII	Ananyeva et al., 2008
LRP1 and LRP2	Connective tissue growth factor	Gerritsen et al., 2010; Kawata et al., 2012
LRP1 and LRP2	Cytochrome C	Lee et al., 2012
LRP1 and LRP2	Cystatin C	Kaseda et al., 2007
LRP1 and LRP2	Epidermal growth factor	Orlando et al., 1998
LRP1 and LRP2	Folate binding protein	Birn et al., 2005
LRP1	Frizzled-1	Zilberberg et al., 2004
LRP2	α-galactosidase	Christensen and Nielsen, 2007
LRP2	Gelsolin	Vargas et al., 2010a
LRP1 and LRP2	Hemoglobin	Gburek et al., 2002
LRP1 and LRP2	Insulin	Orlando et al., 1998
LRP2	Insulin Growth factor I	Carro et al., 2002
LRP1 and LRP2	Lactoferrin	Willnow, 1999
LRP1 and LRP2	Leptin	Dietrich et al., 2008
LRP1 and LRP2	Lipoprotein lipase	Kounnas et al., 1993
LRP2	Liver type fatty acid binding protein	Oyama et al., 2005
LRP2	Lysozyme	Orlando et al., 1998
LRP1 and LRP2	Metallothionein	Klassen et al., 2004
LRP2	Microglobulin	Leheste et al., 1999
LRP2	Myoglobulin	Gburek et al., 2002
LRP2	Neutrophil gelatinase associated lipocalin	Hvidberg et al., 2005
LRP2	Odorant binding protein	Leheste et al., 1999
LRP2	Parathyroid hormone	Hilpert et al., 1999
LRP2	Pancreatitis associated protein 1	Leheste et al., 1999
LRP1 and LRP2	Plasminogen	Kanalas and Makker, 1993
LRP1 and LRP2	Plasminogen activator inhibitory type 1	Stefansson et al., 1995
LRP1 and LRP2	Plasminogen activator inhibitory type 1 urokinase	Moestrup and Verroust, 2001
LRP1 and LRP2	Plasminogen activator inhibitory type 1 tissue plasminoegen activator	Kanalas and Hopfer, 1997; Moestrup and Verroust, 2001
LRP2	Polymyxin B	Moestrup and Verroust, 2001
LRP2	Prolactin	Orlando et al., 1998
LRP2	Pro Urokinase	Stefansson et al., 1995
LRP1 and LRP2	Retinol binding protein	Christensen and Nielsen, 2007
LRP2	Seleno protein P	Olson et al., 2008
LRP2	Seminal vesicle secretory protein II	Ranganathan et al., 1999
LRP2	Sex hormone binding globulin	Hammes et al., 2005
LRP1 and LRP2	Sonic hedgehog protein	Christ et al., 2012
LRP2	Thyroglobulin	Zheng et al., 1998
LRP2	Transcobalamin vitamin B12	Moestrup and Verroust, 2001
LRP2	Transthyretin	Sousa et al., 2000
LRP2	Trichosantin	Chan et al., 2000
LRP2	Vitamin D binding protein	Nykjaer et al., 1999

#### LRP1 expression in the brain

LRP1 is highly expressed in neurons (Andersen and Willnow, 2006), mainly of the entorhinal cortex, hippocampus and cerebellum, activated astrocytes (Rebeck et al., 1995), and microglia (Marzolo et al., 2000). Importantly, LRP1 is further expressed in the central nervous system in different cell types within the neurovascular unit including vascular cells such as brain endothelial cells, vascular smooth muscle cells and pericytes, and it is also expressed in the choroid plexus of the blood-brain barrier (BBB) (Herz and Bock, 2002).

#### LRP1 and signal transduction in the brain

In neurons LRP1 is mainly implicated promoting local catabolism of Aβ. LRP1 is found in the somatodendritic compartment of neurons (Brown et al., 1997), and it can mediate the endocytosis of extracellular ligands in these cells (Makarova et al., 2003). LRP1 also interacts with the neuronally expressed APP (Knauer et al., 1996; Kinoshita et al., 2001) and regulates its proteolytical processing as well as the production of the Aβ peptide (Pietrzik et al., 2002), a process that is of central importance for the pathogenesis of AD. Direct binding of LRP1 to the APP has been shown to affect endoproteolytic processing of APP to increase the production of Aβ42 peptides (Rebeck et al., 2001), which are the major constituent of amyloid plaques (Iwatsubo et al., 1994). LRP1 can promote Aβ production by altering the processing of APP through interactions via the Kunitz protease inhibitor (KPI) domain. Although the non-KPI-APP isoform can weakly bind to LRP1 through cytoplasmic adaptor proteins, such as FE65 (Pietrzik et al., 2004), APP695 processing may not be significantly influenced by LRP1. Rather than promoting local catabolism of Aβ in neurons, LRP1, which is expressed in the neurovascular unit and the choroid plexus might also mediate export of Aβ across the BBB and brain cerebrospinal fluid barrier (BCSFB) (Deane et al., 2004a,b; Zlokovic, 2004). In brain endothelial cells and epithelial cells of the choroid plexus LRP1 may bind directly to Aβ1-40 and export it across the BBB and BCSFB (Deane et al., 2004a,b; Fujiyoshi et al., 2011). In this context, LRP1 and P-glycoprotein (P-gp) have been implicated in Aβ efflux (Shibata et al., 2000; Hartz et al., 2010; Katsouri and Georgopoulos, 2011). LRP-1 is located on the abluminal endothelial cell membrane, whereas P-gp is located on the luminal (blood-facing) surface. The receptor for advanced glycation end products (RAGE), also located on the luminal side of the endothelium, has been linked to Aβ influx (Deane et al., 2003; Sagare et al., 2011a,b).

LRP-1 is also playing other important roles in the central nervous system (Fuentealba et al., 2009, 2010), especially in neurons where it is highly expressed and where it interacts with numerous neuronal proteins such as the postsynaptic density protein 95 (PSD-95) and the N-methyl-D-Aspartate (NMDA) receptor. LRP1 has been found to regulate calcium influx into neurons following stimulation with the glutamate receptor agonist NMDA (Qiu et al., 2002). The molecular mechanism that underlies this effect has not yet been identified. However, the possibility that LRP1 might modulate the functions of neuronal synaptic proteins is in agreement with the results by May et al. (2002); using primary cultured neurons, they showed that LRP1 is present in close proximity to the NMDA receptor in dendritic synapses and can be co precipitated with both NMDA receptor subunits and the postsynaptic density protein PSD-95. Moreover, treatment with NMDA, but not dopamine, reduces the interaction of LRP1 with PSD-95, indicating that LRP1 participates in transmitter-dependent postsynaptic responses, where it would be able to modulate the conductance of neuronal ion channels. Moreover, LRP1 has been shown to regulate calcium signaling *in vitro* (Bacskai et al., 2000), an important second messenger during glutamate neurotransmission. The active form of α2M, an LRP2 ligand, inhibits the calcium-dependent NMDA responses and the expression of NMDA receptors, through a signaling pathway involving LRP1 (Qiu et al., 2002). In fact, mice lacking LRP1 in neurons exhibit a severe mobility disorder, hyperactivity and premature death (May et al., 2004)

An interesting function of LRP1 in neurons is its ability to bind prion protein (PrP) in neurons. Several papers argue that LRP1 controls the surface and biosynthetic trafficking of normal cellular prion protein (PrPC) in neurons. The trafficking of PrPC is believed to control its conversion to the altered conformation (designated PrPSc) associated with prion disease. It was demonstrated that LRP1 is able to associates with PrPC during its endocytosis and is functionally required for this process. Experimentally it was showed that PrPC and LRP1 can be co-immunoprecipitated from the endoplasmic reticulum (ER) in normal neurons. The N-terminal domain of PrPC binds to purified human LRP1 with nanomolar affinity, even in the presence of 1 mM of the LRP-specific chaperone, (RAP) (Taylor and Hooper, 2007).

For infectious prion protein (designated PrPSc) to act as a template and convert normal PrPC to its distinctive pathogenic conformation, the two forms of PrP must interact closely. Interestingly, the neuronal receptor, that rapidly endocytoses the PrPC, is the LRP1. Parkyn et al. (2008) showed here that on sensory neurons LRP1 is also the receptor that binds and rapidly endocytoses smaller oligomeric forms of infectious prion fibrils, and recombinant PrP fibrils. When PrPSc is endocytosed, PrPSc fibrils are routed to lysosomes, rather than recycled to the cell surface with PrPC. Thus, although LRP1 binds both forms of PrP, it traffics them to different destinations within sensory neurons. The binding to ligand cluster 4 should enable genetic modification of PrP binding without disrupting other roles of LRP1 essential to neuronal viability and function, thereby enabling *in vivo* analysis of the role of this interaction in controlling both prion and LRP1 biology (Parkyn et al., 2008; Jen et al., 2010).

However, the most important function of LRP1 in neurons is the major role in the transport and metabolism of cholesterol associated with ApoE-containing lipoproteins. Cholesterol is an essential component of neuronal membrane and myelin sheaths, and is crucial for synaptic integrity and neuronal function (Pfrieger, 2003). Reduced synthesis and increased need for cholesterol by neurons in adult brains require active cholesterol transport to these cells to support synaptic functions and repair (Bu, 2009). Addition of cholesterol to cultured neurons strongly enhances the number and efficacy of synapses in a ApoE dependent manner (Mauch et al., 2001).

Brain ApoE particles, produced primarily by astrocytes, deliver cholesterol and other lipids to neurons via ApoE receptors (ApoER), which belong to the low-density lipoprotein receptor family (Herz and Bock, 2002; Bu, 2009). ApoE promotes the neuronal uptake of cholesterol via LRP1.

In addition to transporting ligands to the cells, ApoE receptors also mediate cellular signaling by binding a variety of extracellular ligands and intracellular adaptor proteins (Herz and Chen, 2006). The best characterized signaling pathway is triggered by Reelin and mediated by type 2 of ApoER. It is well described that Reelin signaling is crucial for neuronal migration (Trommsdorff et al., 1999), dendritic spine development (Niu et al., 2008) and synaptic plasticity (Beffert et al., 2005).

The association of ApoE with AD is very well described in the literature. To since 1990's the ApoE was immunochemically localized to the senile plaques, vascular amyloid, and neurofibrillary tangles of AD. The gene for ApoE is located on chromosome 19q13.2, within the region previously associated with late-onset familial AD. Analysis of ApoE alleles in Alzheimer disease and controls demonstrated that there was a highly significant association of ApoE type 4 allele (APOE-epsilon 4) and late-onset familial Alzheimer disease. Although biochemical evidence suggests that ApoE interferes with Reelin binding to ApoE receptors (D'Arcangelo et al., 1999), the relationship between the two proteins is still not clear. In neurons, ApoE isoforms differentially affect several signaling cascades through ApoE receptors, including increased phosphorylation of disabled 1 (Dab1), activation of the extracellular signal-regulated kinase 1/2 (ERK1/2) pathway and inhibition of the c-Jun N-terminal kinase 1/2 (JNK1/2) pathway (Hoe et al., 2005). ApoE4, but not ApoE3, significantly increases resting calcium, calcium response to NMDA and neurotoxicity in a LRP1 dependent manner (Qiu et al., 2003). Interestingly, ApoE3/lipoprotein affords greater protection from apoptosis than ApoE4/lipoprotein via LRP1-mediated signaling that involves activation of protein kinase Cδ (PKCδ) and inactivation of glycogen synthase kinase-3β (GSK3β) (Hayashi et al., 2007). The implication of LRP1 and its ligands in the pathogenesis of AD is very well described (Vasquez-Higuera et al., 2009).

Several evidences implicated LRP in the pathogenesis of AD. Another interestingly option is the relationship between extracellular matrix and the LRP signaling. Heparan sulphate proteoglycans (HSPGs) are abundant cell surface receptors that interact with a variety of ligands through electrostatic interactions (Poon and Gariepy, 2007). HSPGs found on the surface of almost all mammalian cells are members of the glycosaminoglycan family of polysaccharides and are involved in a large number of biological processes. In neurodegenerative diseases several HSPGs co-localize with senile plaques and cerebral amyloid angiopathy (van Horssen et al., 2003). Heparin and heparan sulphate are able to modify the properties of growth factors activities (Spuch et al., 2004, 2006), in fact theses proteoglycans are able to bind to Aβ (Brunden et al., 1993) and attenuate neurotoxicity and inflammatory activity of Aβ, suggesting a potentially important role for HSPG in cellular metabolism of Aβ (Bergamaschini et al., 2002). In addition, LRP1 and HSPG are part of an immunoprecipitable complex at the cell surface to mediate lipid metabolism (Wilsie and Orlando, 2003). Aβ may initially bind to the HSPG sites on the surface of the complex and then may undergo endocytosis via LRP1, in a process analogous to another LRP1 ligand Internalization of ApoE/lipoprotein particles is partially dependent on the HSPG and LRP1 complex (Mahley and Ji, 1999), suggesting a cooperative function for these ApoE receptors at the neuronal and astrocytes cell surface (Kanekiyo et al., 2011).

#### Intracellular domain (ICD)

LRP-1 has also been shown to interact with scaffolding and signaling proteins via its intracellular domain in a phosphorylation-dependent manner and to function as a co-receptor partnering with other cell surface or integral membrane proteins. LRP-1 is thus implicated in two major physiological processes: endocytosis and regulation of signaling pathways, which are both involved in diverse biological roles including lipid metabolism, cell growth/differentiation processes, degradation of proteases, and tissue invasion. The embryonic lethal phenotype obtained after target disruption of the LRP-1 gene in the mouse highlights the biological importance of this receptor and revealed a critical, but yet undefined role in development. Tissue-specific gene deletion studies also reveal an important contribution of LRP1 in the central nervous system, in vascular remodeling (especially brain vascular endothelium), foam cell biology, and also in the molecular mechanisms of atherosclerosis.

As the case for numerous receptor and membrane proteins, the extracellular domain (ECD) of LRP1 can be cleaved by cell surface proteases and subsequently released into extracellular space or the circulation (plasma or CSF) (Zlokovic, 2011). This cleaved form of LRP1 contains the α-chain of about 515 kDa and a fragment of β-chain of about 55 kDa, demonstrating that the cleavage occurs close to the plasma membrane (May et al., 2003). Enzymes that can mediated this cleavage include the neuronal BACE1 (von Einem et al., 2010) and metalloproteinase (Selvais et al., 2011). The physiological mean of LRP1 soluble form is not certain, but since the soluble form can still bind most of the LRP1 ligands and thereby reduce their endocytoses by cellular LRP1, the soluble fragment may serve to quench extracellular ligand interaction with cell or regulate their intracellular trafficking. Zlokovic's group has identified the LRP1 such as a major Aβ-binding protein in plasma. This soluble receptor may bind 70–90% of the Aβ that circulates in peripheral blood and seems to function as a peripheral sink for Alzheimer's disease causing brain Aβ. Using a mouse model of Alzheimer's disease, the authors demonstrated that boosting the capacity of the sink by administering a form of soluble LRP1, reduces brain amyloid plaque load and improves learning and memory. They extend these results by demonstrating that patients with AD have depressed plasma soluble LRP1 levels.

There is growing evidence that proteolytic degradation of membrane spanning regulatory proteins is involved in a variety of important trans-membrane signaling processes. This mechanism of regulated intramembrane proteolysis (RIP) enables them to respond to extracellular signals. γ-secretase may play a central role in a signaling paradigm that has been termed RIP. RIP processing is described in different receptors such as p75NTR, ErbB4, APP, Notch and also LRP1 and LRP2, by allowing ICD to translocate to the nucleus (Hass et al., 2009; Spuch and Navarro, 2010a,b; Spuch and Carro, 2011; Groot and Vooijs, 2012). Alternatively, RIP may turn off signaling events in which the transmembrane anchored protein is responsible for signaling and cleavage terminates the signal.

An extremely important point in regards to RIP, largely ignored by most investigators, is that the fate of any ICD is dependent on its N-terminus that dictates the stability of the cleaved products. According to the N-end rule, only ICDs whose N-terminus evades ubiquitination escape degradation, whereas fragments beginning in other residues undergo rapid proteasomal degradation (Tasaki and Kwon, 2007).

This potential mechanism in LRP1 regulation involves the cleavage of the transmembrane domain of the LRP1 β-chain by RIP. The released fragment (LRP1-ICD) of 12 kDa might thus translocate to the nucleus where it can regulate the transcription of target genes (Derocq et al., 2012). LRP1 following PKC activation and metalloproteinase-induced shedding of the (ECD), the LRP1-ICD is released from the membrane by γ-secretase. This cytoplasmic fragment may have functions in the cytoplasm or in the nucleus, including transcriptional regulation. Although the LRP1-ICD functions are still unknown, recently, one potential target of the LRP1-ICD has been identified. Lipopolysaccharide (LPS) increases the proteolytic processing of the ectodomain of LRP1, which results in the γ-secretase-dependent release of the LRP1-ICD from the plasma membrane and its subsequent translocation to the nucleus, where it interacts with and represses the interferon-γ promoter (Zurhove et al., 2008).

The LRP1-ICD fragment contains numerous motifs that have been implicated in numerous signaling pathways: Two NPXY motifs, where the distal motif is contiguous with a YXXL motif, and two dileucine motifs. The YXXL motif is presumably the most important one mediating LRP1 endocytosis (Li et al., 2000). However, both NPXY motifs can bind and interact with numerous cytosolic proteins such as, DAB1, FE65, JIP1, PSD-95, ShcA or CED-6/GULP (Berger et al., 2010; Boucher and Herz, 2011). *In vitro* studies have shown that the LRP1-ICD can colocalize with the histone acetyl transferase Tip60 in the nucleus (Kinoshita et al., 2003), which in turn can regulate transcription upon APP cleavage (Baek et al., 2002) suggesting that the LRP1-ICD might be able to regulate the transcriptional activity of the APP-Tip60 complex, and thus have a more general function as a regulator of transcription.

### Low density lipoprotein receptor-related protein-2 (LRP2)

**Figure 2:**
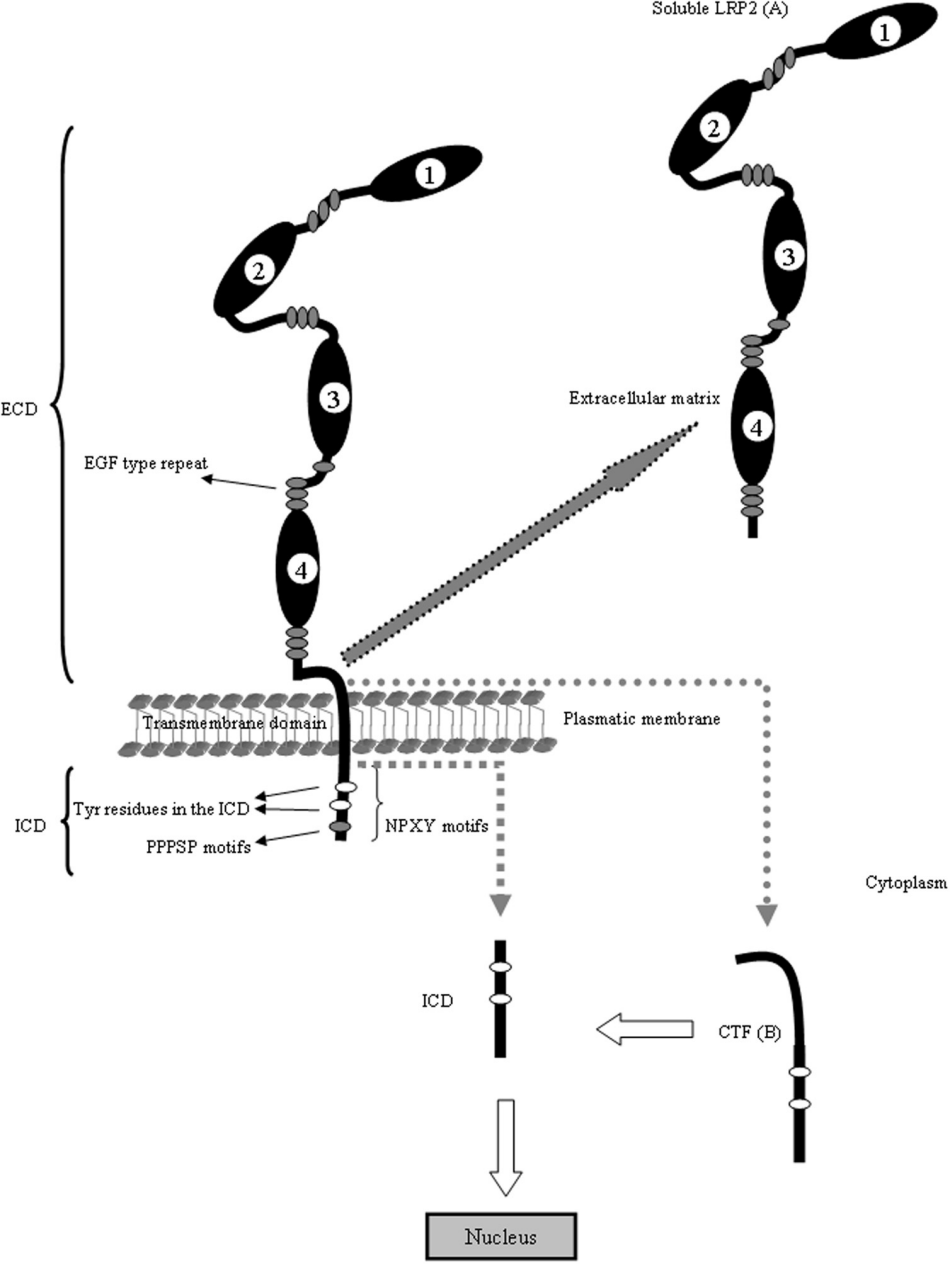
**LRP2 is structurally similar to LRP1.** The ECD of LRP2 contains four clusters (1–4) of lipoprotein receptor ligand-binding repeats, growth factor repeats, an EGF repeat, and YWTD spacer regions. Interestingly, the second cluster has been identified as a common binding site for several ligands including Apo E, Apo M, retinol binding protein and transthyretin. The fourth cluster has been identified as binding site for Aβ. The LRP2 is also proteolytically cleaved to generate two subunits: **(A)** the N-terminal (soluble LRP2) containing the ligand-binding domains and **(B)** the C-terminal subunit containing an extracellular fragment (CTF), the transmembrane spanning domain and the ICD. The ICD contains diverse potential endocytosis and signaling motifs. The ICD binds adaptor proteins important for LRP2-mediated endocytoses, such as Dab2 and is able to induce intracellular events such as RIP signaling. The cytoplasmic domain of LRP2 has several putative internalization motifs, including one dileucine and three NPxY motifs. In addition, it contains two proline-rich sequences, one PDZ terminal motif, several putative protein kinase C and casein kinase II phosphorylation motifs as well as one protein kinase A phosphorylation motif. Under basal conditions, these motifs contribute little to the phosphorylation of the LRP2 cytoplasmic domain. Although there are few evidences about the cytoplasmic regulation of LRP1 and LRP2, the mechanism is conserved in the gene evolution and probably the regulation and trafficking of both receptors in neurons are very similar. With arrows is indicating the cleavage events in the molecule and the resulting fragments.

LRP2, also called megalin, is one of the largest cell surface glycoproteins present in vertebrates, is a transmembrane protein with a non-glycosytaled molecular weigh of 517 kDa (Saito et al., 1994; Chowdhary et al., 1995; Spuch and Navarro, 2010a,b). This receptor is structurally very similar to LRP1. LRP2 is composed of a large ECD consisting of four cysteine-rich complement-type ligand binding repeats, responsible for ligand binding (Table 1), that binds to the chaperone RAP (receptor associated protein) for its folding in the (ER) (Bu and Marzolo, 2000). The repeats are separated from each other by β-propeller domains (Saito et al., 1994), structured by repeats of YWTD flanked by EGF-like modules, that are generally important for the proper receptor folding in this family of proteins (Culi et al., 2004; Lighthouse et al., 2010) as well as for the dissociation of their ligands in the acidic endosomal compartment (Jeon et al., 2001). In addition, LRP2 contains one transmembrane domain that targets it to membrane domains rich in cholesterol and glycosphingolipids (Marzolo et al., 2003) and is also a substrate for the γ-secretase complex (Zou et al., 2004). Among these are three NPXY motifs that have been linked to LDLR and LRP1 internalization mediated by clathrin, recycling from the endosomal compartment to the plasma membrane and basolateral distribution (Donoso et al., 2009). However, the roles of these motifs have not been clearly defined for LRP2 (Figure 2).

#### LRP2 expression in the brain

In the healthy brain, the expression of LRP-2 is classically published in ependymal cells lining the ventricular wall, capillaries and choroid plexus (Zheng et al., 1994; Chun et al., 1999; Carro et al., 2005). The expression is principally restricted to epithelial cells, specifically at the apical surface (Willnow, 1999). Interestingly, despite the presence of some putative basolateral sorting motifs in the cytoplasmic domain of LRP2, its apical localization depends mainly on sorting information present in this domain of the receptor because its addition to a reporter protein that lacks sorting information drives trafficking of the reporter to the apical surface of polarized epithelial cells (Marzolo et al., 2003) However, during the last years several papers were published describing the expression of LRP2 within peripheral and central nervous system. The first evidences for LRP2 localization in the central nervous system were described through developmental studies. It is well known that genetic deficiency of LRP-2 or inactivation of the *lrp2* gene leads to holoprosencephalic phenotype, characterized by abnormal development of the forebrain, absence of olfactory apparatus and cranio-facial malformations (Assemat et al., 2005). A novel mutation in *lrp2* gene that causes an enlarged cortex, abnormalities in the dorsal diencephalon further hypertrophy of the choroid plexus of the third ventricle was also identified (Zarbalis et al., 2004).

Within the central nervous system, further ependyma of the choroid plexus (Spuch and Navarro, 2010a,b), LRP-2 is widely expressed on neurons (LaFerla et al., 1997) and astrocytes (Bento-Abreu et al., 2008). In astrocytes this receptor is required for albumin binding and internalization into astrocytes inducing synthesis of neurotrophic factors by the neighbouring neurons (Bento-Abreu et al., 2009). Other observations identified that LRP-2 is present in retinal ganglion cells and in astrocytic processes of young and adult rats. In these cells, LRP-2 interacted with metallothionein-IIA allowing the activation of different intracellular signaling pathways involved in neuroprotection (Fitzgerald et al., 2007). In this context, studying the role of metallothionein as a neuroprotective factor and ligand of LRP-2, it was described the expression of LRP-2 in cerebellar granule cells being mediator of the neuroprotective action of metallothionein (Ambjorn et al., 2008). Due to the growing evidence that LRP-2 expression in the brain is not restricted to tight-junction epithelia, oligodendrocytes and glial cells, our group recently published the broad expression of this endocytic receptor in different neuronal populations of the brain. In brain samples from healthy humans, monkeys, pigs, mice and rats we demonstrated LRP2 localization in different neuronal populations from cerebral cortex, hippocampus, striatum, thalamus, olfactory bulb and cerebellum (Alvira-Botero et al., 2010). Interestingly, in brain tissues from patients diagnosed with Alzheimer's disease, LRP-2 expression has been immunohistochemically detected in neurons, even being up regulated in damaged neurons (LaFerla et al., 1997).

#### LRP2 and signal transduction in the brain

LRP2 is an endocytic receptor which binds its extracellular ligands before an endocytic uptake. The activation of different signaling pathways is due to LRP2 dependent internalization of a number of ligands representing a wide variety of molecules, including lipoproteins, hormones, vitamin-binding proteins and drugs (Table 1). The signaling functions in the cytoplasm are controlled by their interaction with adaptor proteins that recognize specific motifs within the cytoplasmic domains of LRP-2. There are evidences linking LRP2 endocytosis to non-clathrin mediated pathways involving trafficking proteins such as the small GTPase Arf6 (Wolff et al., 2008) and caveolin 1 (Bento-Abreu et al., 2009). Two cytoplasmic proline-rich sequences and a PDZ-binding motif have been implicated in the direct and indirect interaction of LRP2 with cytoskeletal and cytosolic scaffold and signaling proteins, such as GIPC/ synectin, megalin-binding protein, ANKRA, myosin VI, SKIP, Disabled 2 (Dab2) and APPL1 (Rader et al., 2000; Patrie et al., 2001; Larsson et al., 2003; Spuch and Navarro, 2010a,b). An interesting feature of LRP2 is that it is constitutively phosphorylated by GSK3 at a PPPSP motif, contained in a distal proline-rich motif of the cytoplasmic tail. This PPPSP motif is the most significant in terms of basal phosphorylation of the receptor, despite the presence of several other consensus phosphorylation sites for PKC, CK-II and PKA and its function is related to the control of LRP2 recycling from the endosomes (Yuseff et al., 2007).

Recent data identify a critical role for LRP2 in SHH signaling and reveal the molecular mechanism underlying forebrain anomalies in mice and patients with *Lrp2* defects. This group identified LRP2 as a component of the SHH signaling machinery in the rostral diencephalon ventral midline. LRP2 is acting as an apical SHH-binding protein that sequesters SHH in its target field and controls internalization and cellular trafficking of SHH/patched 1 complexes (Christ et al., 2012).

In the brain, LRP2 participates in endocytosis and internalization of ApoE, APP and Aβ peptide (LaFerla et al., 1997). The first signaling event described for LRP2 was suggested by Strickland's group showing that LRP2 is responsible for soluble-APP endocytosis (Kounnas et al., 2008). Recently, our group published in cortical and hippocampal neurons that LRP2 is able to form a macromolecular complex together with APP and Fe65 acting as a negative regulator of neurite branching and as mediator of Aβ neurotoxicity (Alvira-Botero et al., 2010). In another study with granule cells from cerebellum described as metallothionein induced neuronal differentiation, survival and initiate signal transduction pathways resulting in neurite outgrowth by binding of LRP2 (Ambjorn et al., 2008). In spite of the poor information available it seems that the activation of LRP2 upon ligand binding mediates neurite outgrowth and apoptosis.

Also, it was demonstrated that LRP2, a receptor implicated in BMP4 clearance is specifically expressed in ependymal cells of the lateral ventricles in the adult brain. Intriguingly, expression is restricted to the ependyma that faces the stem cell niche. Expression is not seen in ependyma elsewhere in the lateral ventricles or in the dentate gyrus, the second major neurogenic zone of the adult brain. This group further showed that lack of LRP2 expression in adult mice results in impaired proliferation of neural precursor cells in the subependymal zone resulting in decreased numbers of neuroblasts reaching the olfactory bulb. Reduced neurogenesis coincides with increased BMP4 expression and enhanced activation of downstream mediators phospho-SMAD1/5/8 and ID3 in the stem cell niche. These findings suggest a novel mechanism whereby LRP2-mediated catabolism of BMP4 in the ependyma modulates the microenvironment of the subependymal zone and enables adult neurogenesis to proceed (Gajera et al., 2010).

#### Intracellular domain

Similarly to what described for LRP1, LRP2 is also able to initiate signaling events related with RIP processing in a manner similar to that described for APP and Notch. This receptor undergoes proteolytic shedding of the ECD by a metalloproteinase. It has recently been shown that the cytoplasmic tail of LRP-2 can be processed intramembranously by a γ-secretase activity that releases its ICD-LRP2 (Biemesderfer, 2006). Following the RIP processing in the cytoplasmic domain of LRP2, this receptor also undergoes proteolytic shedding of the ECD by a metalloproteinase, generating truncated forms, also named soluble-LRP2, corresponding with the 4-loop of LRP-2 (Ishida et al., 2006). The COOH-terminal fragment is in turn released from the membrane by γ-secretase activity acting at a cleavage site within the proteins membrane spanning domain. Once released, the COOH-terminal domain traffics to the nucleus where, through interaction with transcription factors, it controls expression of target genes (Ebinu and Yankner, 2002). However, related to COOH fragment of LRP-2 nothing is known about its regulation in neurons. Our group found LRP2-ICD in rat hippocampal neurons *in vitro* (unpublished data). Furthermore, the function of these events in the context of the brain is completely unknown. We suggest that LRP2-ICD could be regulated by LRP2 ligands and may be involved in gene transcriptions and apoptosis events, although more investigations are necessary to discover relevant facts about LRP2-ICD in neurons. The unique evidence about the function of LRP2-ICD was described in kidney, where it seems likely that RIP of LRP-2 is part of a more complex molecular pathway that ensures LRP2 expression at some necessary physiologic level (Li et al., 2008).

## LRP-1/LRP-2 roles in AD

Advancing age is a major risk factor for many neurodegenerative disorders, and the major risk factor for AD, a disease characterized by progressive memory and cognitive loss (Selkoe et al., 2012). The most accepted hypothesis for the mechanism of brain injury in AD is the “amyloid cascade,” comprising amyloid accumulation in the brain, the formation of toxic oligomeric and intermediate forms of Aβ peptides, amyloid plaques, inflammation and the induction of neurofibrillary tangles (Jin et al., 2011). There is accumulation of Aβ in both, the normal aging brain and the AD brain, thought to be related to defective Aβ clearance rather than increased Aβ production (Zlokovic et al., 2010). This has recently been shown to be the case in AD. Clearance of this peptide from the brain occurs via active transport at the interfaces separating the central nervous system from the peripheral circulation. Through the BBB and the BCSFB, LRP1 and LRP2, facilitate the clearance of the Aβ peptide that is produced by amyloidogenic processing of the APP, which can form complexes with different LRPs ligands such as ApoJ (also called clusterin) and ApoE (Zlokovic, 2004, 2011; Nuutinen et al., 2009) and ApoE. In addition to these ligands, a neuroprotective role for the Aβ-binding protein called gelsolin (Carro, 2010), that is produced and secreted in the epithelial cells of the choroid plexus (Vargas et al., 2010a), was recently demonstrated. Gelsolin has neuroprotective functions in controlling the Aβ-induced production of NO and apoptosis as well as cytoskeletal disruptions in the epithelial cells of the cerebrospinal fluid barrier (Vargas et al., 2010a).

Several lines of evidence have implicated LRP and LRP ligands in the pathogenesis of Alzheimer's disease (Nieoullon, 2011). First, LRP is a major neuronal receptor for ApoE/lipoprotein, and the epsilllon4 allele of ApoE is a strong genetic risk factor for late-onset AD. LRP-mediated brain metabolism of ApoE/lipoprotein can also influence the metabolism of cholesterol, which has been suggested to contribute to the pathogenesis of AD by regulating Aβ metabolism. Second, immunoreactive staining of several LRP ligands (e.g., ApoE, α2 M, and tissue factor pathway inhibitor) as well as LRP itself has been detected in senile plaques. Third, some genetics studies have suggested that LRP1 and LRP2 are linked to AD and cerebral amyloid angiopathy (Ballatore et al., 2007). Two recent genome-wide association studies have reported that PICALM (phosphatidylinositol binding clathrin assembly protein) and ApoJ (also known as clusterin) are the only two AD susceptibility genes (Harold et al., 2009). However, this year it was published a study where the 10 most promising late-onset AD susceptibility genes identified through several recent large GWAS (APOE, CLU, PICALM, CR1, BIN1, ABCA7, MS4A6A, CD33, CD2AP, and EPHA1). This study has been identified curiously, apart from the APOE locus which showed compelling evidence of association with risk on human life span, none of the other gene loci demonstrated significant evidence of association (Shi et al., 2012). However, last studies of Carro's group demonstrated three new polymorphisms in the genes PLA2G3, IGF-I and LRP2 associated with AD in a Spanish population (Martínez-García et al., 2010; Vargas et al., 2010b, 2011).

Together these observations indicate that LRP can participate in AD pathogenesis by altering the catabolism of LRP ligands (e.g., IGF-I, ApoE/lipoprotein/cholesterol, tPA, and α2M) and/or influencing Aβ metabolism and accumulation (Carro et al., 2002, 2005, 2006). Further, it has been shown that the LRP1 and LRP2 cytoplasmic C-terminal domain interact with APP cytoplasmic domain via FE65, which in turn influences APP processing and Aβ generation (Pietrzik et al., 2004; Alvira-Botero et al., 2010).

Recent findings have revealed the roles of γ-secretase and LRP1 in the inhibition of the inflammatory response suggesting that both proteins may serve as potential therapeutic targets for the modulation of inflammation (Zurhove et al., 2008). Furthermore, none is known about the speculative effect of LRP2 in the neuroinflammation. Probably, the modulation of LRP1 and LRP2 in the different cells of the neurovascular unit could be new therapeutic strategy.

## Conclusions

In summary, we have reviewed recent evidence suggesting that LRP1 and LRP2 have a major role in regulating brain and systemic clearance of A^β^. Since the discovery of both LRPs as important endocytic receptors present in the brain endothelium and epithelial cells of the choroid plexus several new ligands and functions for these proteins have been uncovered. Recent findings in LRPs functions in the brain cells (astrocytes, glial cells and neurons) and their role in the internalization of different molecules, open the possibility that these receptors can be used as a target and regulator of signal transduction pathways, as well as the emergence of its roles in neurodegeneration and regeneration processes and in chronic and genetic diseases. The modulation of LRP1 and LRP2 in the different cells of the neurovascular unit could be new strategy therapeutic. However, as a note of caution, the development of LRPs-based therapies for neurodegenerative diseases requires careful toxicity and safety monitoring of unwanted potential side effects given that LRPs participate in multiple control systems in the body (cellular transport in different organs, anticoagulation process and inflammation).

### Conflict of interest statement

The authors declare that the research was conducted in the absence of any commercial or financial relationships that could be construed as a potential conflict of interest.
